# REVISITING THE TEACHING NURSING HOME: PRAGMATIC EVALUATION IN A REAL-WORLD SETTING

**DOI:** 10.1093/geroni/igad104.1545

**Published:** 2023-12-21

**Authors:** Howard Degenholtz

**Affiliations:** University of Pittsburgh, Pittsburgh, Pennsylvania, United States

## Abstract

Revisiting the Teaching Nursing Home (RTNH) is a renewal of the Teaching Nursing Home model pioneered in the 1980s, which found that creating partnerships between schools of nursing (SONs) and nursing homes (NHs) improved clinical outcomes. In its first two years, the RTNH initiative has fostered partnerships between three SONs and four NHs in Pennsylvania, and is planning to expand. The program is implementing the Institute for HealthCare Improvement Age-Friendly Health System “4Ms” quality improvement framework through an online learning network. The 4M quality improvement framework focuses on four evidence-based elements: Mobility, Mentation, Medication, and What Matters. In addition, the participating SONs are incorporating the 4Ms into curricula and clinical rotations. The evaluation will examine both implementation and outcomes for SONs and NHs. Measures include student competency, staff satisfaction and burnout, clinical process of care and resident outcomes. While some resident level measures can be extracted from the Minimum Data Set (MDS), others (deprescribing, rehabilitation goals, preference sensitive care) require access to the electronic medical record (EMR). The goal of this presentation is to describe the methodology for measuring the wide range of outcomes and to examine the performance of a synthetic comparison group to study overall quality using CASPAR data. We selected 1,764 similarly sized, non-profit NHs and conducted bivariate, and propensity score analysis to determine if the participating NHs were different on any observable characteristics. The findings suggest that the participating NHs were not statistically significantly different in terms of overall quality rating, staffing, or inspections.

